# Psychometric properties and cross-cultural adaptation of the Indonesian version of the Brief COPE in a sample of advanced cancer patients

**DOI:** 10.1371/journal.pone.0275083

**Published:** 2022-11-28

**Authors:** Nurul Huda, Yen-Kuang Lin, Malissa Kay Shaw, Yu-Ying Hsu, Hsiu-Ju Chang

**Affiliations:** 1 Nursing Faculty, Universitas Riau, Pekanbaru, Indonesia; 2 Graduate Institute of Athletics and Coaching Science, National Taiwan Sport University, Taoyuan City, Taiwan; 3 Graduate Institute of Humanities in Medicine, Taipei Medical University, Taipei, Taiwan; 4 School of Nursing, College of Nursing, Taipei Medical University, Taipei, Taiwan; 5 College of Nursing, Department of Nursing, National Yang Ming Chiao Tung University, Taipei, Taiwan; 6 College of Nursing, Efficient Smart Care Research Center, National Yang Ming Chiao Tung University, Taipei, Taiwan; Mugla Sitki Kocman Universitesi, TURKEY

## Abstract

The Brief COPE Inventory has been proven as acceptable psychometric properties to examine coping strategies among cancer patients. However, most psychometric testing studies have been carried out in Western countries, raising concerns about the properties’ relevance and applicability in other cultural contexts. This study aimed to present psychometric properties of the Brief COPE in a sample of patients with advanced cancer in Indonesia. Specifically, we intended to examine the factorial structure and the measure’s validity and reliability. This study included 440 patients from the original study who completed the Indonesian version of Brief COPE. We used exploratory factor analysis and confirmatory factor analysis to assess factor structure and evaluate the structural model fit, respectively. Reliability was demonstrated by internal consistency represented by Cronbach’s alpha coefficient. The factor analysis identified a 21-items scale with 5-factors (avoidance, religion and acceptance, social support coping, problem solving and distraction). Confirmatory factor analysis demonstrated a good model fit. For the whole scale and its subscales Cronbach’s alpha coefficients were acceptable signifying good reliability. Convergent, divergent validity and contrast group comparison were evidenced by significant correlations among subscales and the other instruments used. This study shows that the Indonesian version of Brief COPE is a reliable and valid instrument to measure coping in advanced cancer patients and is ready for use amongst this population in the Indonesian cultural context.

## Introduction

Globally cancer is one of the leading causes of death. In general, the incidence rate of cancer in developing countries is lower than economically developed countries, however, case fatality is higher indicating a more unfavourable survival rate [1]. In Indonesia, cancer ranks as the seventh most prevalent cause of mortality and more than 70% of sufferers are diagnosed in an advanced stage [2]. Although the advancement of cancer care has improved, advanced cancer patients (ACP) may still experience various physical and mental disturbances [3].

A previous study found that almost half of ACP meet criteria for psychiatric disorders, but less than 10% are referred for further psychological help [4]. Left untreated, these mental health problems may lead to negative consequences such as treatment non-compliance, lower quality of life (QoL) and poor survival [5]. Research on advanced cancer found that appropriate coping strategies are correlated with better mental health and QoL [6]. In addition, elaborating on the structuring of coping strategies when delivering interventions has been proven effective in decreasing symptom severity and ameliorating the burden of disease [6]. However, a major conceptual issue with these approaches is the healthcare provider largely ignoring the coping strategies that ACP use, which could lead to mental health deterioration [7].

Coping has been defined as cognitive and behavioural efforts to handle particular demands, both external and internal, that surpass one’s available resources [8]. The coping strategies used depend on individuals’ appraisal of the situation that they face, and may function to protect or harm their wellbeing [9]. Previous literature found that coping in ACP is the result of evolution where it does not only represent the process of establishing an appropriate strategy to maintain their wellbeing but additionally how the strategies utilized transform as diseases progress or illness experiences develop [10]. Therefore, interventions to help ACP adopt proper coping strategies along their illness trajectories may have beneficial impacts, but their design requires a cultural awareness of the diversity of coping strategies and reliable means to measure such strategies.

Several instruments have been formulated for measuring coping. The literature most often mentions the Brief Coping Orientation to Problems Experienced Inventory (COPE) purposed by Carver [11]. This instrument has also been employed for measuring coping in advanced cancer studies in multiple sites as well as various populations, which proves empirical support to utilize this instrument in this population [12]. It has been proven as acceptable psychometric properties and used extensively to examine various coping strategies and psychological outcome in cancer survivors and ACP [13, 14]. However, the Brief COPE’s structure appears to be substantially unstable as results largely depend the method of factor analysis utilized [15]. As suggested by Carver (1997) [11], researchers should use the Brief COPE flexibly and creatively as needed to justify an exploratory analysis. Thus, they can empirically establish the way in which their sample’s data will be analysed. In addition, most versions of Brief COPE have been developed in western countries which have distinct cultures, beliefs and norms. For example, ACP from Nordic countries more often used emotional-focused coping [16] while ACP in Indonesia more commonly employed problem-focused coping [17]. Furthermore, family and spiritual support were essential to the ability of Asian ACP to cope and recover whereas acceptance was the most prevalent coping strategy used among ACP in six European countries [12]. These variations raise concerns about the relevance and applicability of Brief COPE in other cultural contexts such as in Indonesia.

The development of the Indonesian version of Brief COPE involved bilingual experts translating it into the Indonesian language and then back translating it into English. A previous research team then compared the original and back translated versions of Brief COPE and revised some items based on experts’ judgments. Although the reliability of this translation is acceptable (Cronbach’s **α** = 0.825), proper psychometric properties such as intraclass correlation, validity and factor analysis have not been yet established. In addition, the original sample only consisted of 70 participants most of which were in an early stage of cancer, meaning the questionnaire had limited use in a broad setting [18]. Given this, there is a need for validating scales to measure coping strategies of ACP in Indonesia and explore its psychometric properties. Moreover, given the broad use of Brief COPE in different populations, we desire to explore the factor structure using confirmatory factor analysis (CFA) because the majority of previous studies applied exploratory factor analysis (EFA) which limit the use of goodness of fit to see if data fit a hypothesized measurement model driven by theory. Therefore, the purpose of the current study was to evaluate the psychometric properties of the Indonesian version of Brief COPE in a sample of ACP in Indonesia. Specifically, we aimed to examine the factorial structure of this measure and evaluate the validity and reliability of the construct.

## Methodology

### Study design and sample characteristics

This is a cross-sectional study using secondary data analysis. We used the data from our previous research which investigated mediating of coping in ACP [19]. A total of 440 patients were recruited from May to December 2019 with an overall response rate of 97.8%.

For confirmatory factor analysis, a minimum of 10 observations per variable is necessary to avoid computational difficulties [20]. The sample size meets the required minimum sample size of N = 280 for CFA. However, since we divided the sample for EFA and CFA, the sample size was not enough to run both the EFA and CFA. Therefore we applied bootstrapping method to multiply the sample size [21].

The inclusion criteria stated: age 18 years old and above, confirmed stage III, IV or recurrence cancer by medical oncologist and ability to speak Bahasa fluently [22]. The recruitment of potential participants took place at the outpatient facility of an oncology ward of a general government hospital in Riau Province, Indonesia. The researchers collaborated with the head nurse who identified potential participants who met the inclusion criteria. After patients were initially identified, the principal researcher and two trained research assistants, who were registered nurses with experience working in oncology, collected the data. After agreeing to join the study and providing informed consent, participants were instructed to fill out the questionnaires. From the beginning, patients were fully informed of the study’s purpose and procedures. Research ethics committees in Indonesia (IRB 035/UN.19.5.1.8/UEPKK/2019) and Taiwan (N 201905001) approved the study.

### Instrument development and validation

We engaged in various cultural adaptation steps to validate the Indonesian version of Brief COPE. These included: pilot testing the translated inventory, revising the translation, evaluating content validity, assessing the reliability and validity of the revised inventory, performing EFA to assess the relevance of factor structure and applying CFA.

### Cross-cultural adaptation

Items in the Brief COPE had previously been translated [18]. Permission was obtained to use the Indonesian Brief COPE from the original author. The process of the previous translation was guided by Beaton et al (2000) [23]. The first step of translation was forward translation and involved two translators independently translating the Brief COPE into Bahasa. The translators had a background in psychological and were fluent in English and Bahasa. Once the initial translations were finished, a discussion about any inconsistencies between the two translations took place to form a composite translation. Once an agreement was met, the combined translation was back translated from Indonesian into English by a professional bilingual, native English-speaking translator. This process aimed to reveal whether the Indonesian translation of Brief COPE represents the original version and has the same linguistic content. A detailed discussion considered the conceptual and cultural similarities of the original Brief COPE and the back translation. Following this, the Indonesian version was examined by the translation committee linguistic and interpretation errors, and the final version was agreed upon. A pre-test study was then conducted with 30 participants.

### Content validity testing

To ensure cross cultural adaptation and applicability in our target population, we conducted face validity with a small sample of ACP (N = 10). The respondents were verbally asked by an interviewer to elaborate on what they think each questionnaire item means to determine if the items in the instrument are relevant, reasonable, unambiguous and clear. The face validity results showed that all items were understandable from the respondents’ perspective. A group of bilingual, oncology and mental health experts were then invited to review the revised version in order to assure semantic, conceptual and normative equivalence in a sample of ACP in Indonesia. The experts included one clinical psychiatrist, two medical oncologists, two nursing faculty members (doctoral qualification in mental health and medical surgical nursing), one clinical psychologist and one oncology ward head nurse. The experts had a minimum of five years of experience in their field, held at least a master degree and/or specialist training and could speak and write English and Bahasa fluently.

The Content Validity Index (CVI) was then determined using the I-CVIs and S-CVI/ave. Excellent content validity is obtained if the scores of I-CVI and S-CVI/ave exceed 0.78 and 0.90 respectively [22]. In this study, the I-CVI and S-CVI were 0.85 and 0.97 respectively. The time required for the patients to answer the questionnaires was approximately 5–7 minutes.

### Exploratory Factor Analysis (EFA) and Confirmatory Factor Analysis (CFA)

We first tested CFA based on the two previously specified second order factor models of Brief COPE purposed by Cooper et al. and Meyer et al. [24, 25]. Cooper and colleagues’ model categorizes coping into three groups: emotion-focused coping (emotional support, positive reframing, acceptance, religion and humour), problem-focused coping (active coping, planning and instrumental support) and dysfunctional focused coping (venting, denial, substance use, behavioural disengagement, self-distraction and self-blame). Meyer and colleagues’ model, however, categorizes coping into two groups: adaptive coping strategies (emotional support, positive reframing, acceptance, religion and humour) and maladaptive coping strategies (venting, denial, substance use, behavioural disengagement, self-distraction and self-blame). Due to the poor fit of the two models with the data, an EFA was chosen to establish the factor structure of the Brief COPE. We then applied bootstrapping method to multiply the sample size [21]. The sample was then randomized to distinguish between EFA and CFA. EFA with a principal axis factoring extraction (PAF) method was then conducted. Promax rotation was chosen since all of the coping strategies included in the Brief COPE might have correlation with the others [26]. The minimum item loading was 0.30 [27]. Items were excluded in the case of less loading or multiple loading on several factors.

CFA was used to confirm the factor structure obtained from EFA to support the evidence of construct validity. Several statistical methods were performed to evaluate the fit of the models. One is the chi-square goodness of fit statistics which has statistically significant value when the model is fit. The ratio of value to the degree of freedom with 1< χ2 /df < 5 is considered acceptable. A variety of non-chi-square goodness of fit indices were also tested: comparative fit index (CFI), goodness of fit index (GFI), normed fit index (NFI), root mean squared error of approximation (RMSEA) and root-mean-squared residual (RMR). The recommended acceptable model fit value for CFI, GFI. NFI should be greater than 0.90. For the RMSEA and RMR, the results should be lower than 0.08 and 0.05 respectively to indicate a model fit [28]. All of the collected data were managed and analysed using Statistical Package for Social Sciences (SPSS) version 22.0 and SPSS AMOS version 22.0 (IBM Corporation, Amronk, NY, USA).

### Reliability testing

The reliability was supported by investigating its internal consistency and correlation between test and retest measurements. Cronbach alpha coefficient was used to determine the internal consistency. Cronbach alpha of 0.70 or above is considered acceptable [29]. Furthermore, test-retest reliability was explored by administering the Brief COPE to a sub-sample of 30 ACP two weeks after the initial test. Intra Class Correlation (ICC) and Pearson correlation were examined to support the reliability. ICC’s and Pearson’s correlation coefficient greater than 0.70 are appropriate [30].

### Validity testing

Validity was demonstrated by convergent, divergent, contrasted group comparison and construct validity. Convergent and divergent validities were evaluated by Spearman’s correlation since the variables were not normally distributed. A significant and negative relationship between Brief COPE Inventory and DASS-D, DASS-A as well as DASS-S was used to present the evidence of divergent validity. The Depression Anxiety Stress Scale measures the negative emotional states of depression, anxiety and stress [31].

Evidence of convergent validity was demonstrated by the significant and positive relationship between the Brief COPE and the Functional Assessment of Cancer Therapy-General (FACT-G). FACT-G is an instrument designed to measure QoL of cancer patients along four domains: physical, social, emotional and functional wellbeing [32]. Previous research confirms that ACP that used effective coping strategies may have better QoL [6].

Regarding contrasted group comparison validity, a significantly different score on the Brief COPE and its factors was evaluated by comparing the different level of depression (normal, mild, moderate, severe and very severe). We categorized depression based on the DASS 42 score’s guidelines. The following scores correspond to different severities of depression: 0–9 = normal, 10–13 = mild, 14–20 = moderate, 21–27 = severe, 28–42 = extremely severe. Results showed that 17.70% of respondents had depression symptoms from mild to very severe. ANOVA test was then applied to test group differences on the Indonesian Brief COPE.

### Measures

#### Brief Coping Orientation to Problems Experienced Inventory (Brief-COPE)

Brief-COPE has 28-items that evaluate 14 coping strategies. Each of the strategies comprise 2-items and are rated using a 4-point likert scale. A higher score represents greater coping [11].

### Depression, Anxiety, Stress, Scale (DASS)

DASS measures symptoms of depression, anxiety and stress experienced within the past week and consists of 42-items [31]. Scores exceeding 10, 7 and 14 are considered mild to extreme for depression, anxiety and stress respectively. Good reliability was shown by both the DASS Indonesian version (α = 0.95) and this current study (α = 0.96).

### Quality of life (FACT-G)

FACT-G is a self-report questionnaire that quantifies QoL and consists of 27-items [32]. The Indonesian version of the FACT-G has good reliability, with Cronbach’s α = 0.82 [33]. We utilized the total score of FACT-G which represents overall quality of life for this analysis.

## Results

### Demographic characteristics of respondent

The majority (62.7%) of the respondents were middle-aged adults, 86.6% were female, 73.9% were married and 88.2% were Muslim. Respectively, 48.2% and 70.3% had graduated from senior high school and were not working. Clinical characteristic data showed that the mean score of karnofski performance scale was 73.66. More than 93.4% of the respondents had a solid tumour, 46.59% were in stage 3, 63.4% has been diagnosed with advanced cancer for more than six months, and most participants were only receiving one type of treatment [19].

### Results of CFA based on original subscale and two previous models

First, CFA of the Indonesian version of Brief COPE based on the original 14 subscales was performed. The goodness model of fit was exceptional; however, the validity and reliability were poor. We then tried to analyse the Indonesian Brief COPE Scale with 28-items using specified second order factor models of Brief COPE purposed by Cooper et al. as model 1 and Meyer et al. as model 2 [24, 25]. The test of absolute model fit indicated poor model fit (Table 1).

**Table 1:** Indices of Model 1 and Model 2.

Model	No of factors	X2(df)	Relative x^2^ fit index	CFI	NFI	GFI	RMR	RMSEA
1	3	2853.65 (350)	8.22	.65	.62	.70	.17	.12
2	2	2924.94 (349)	8.38	.64	.62	.69	.17	.13

*Note*. *CFI* = comparative fit index; *NFI =* normed fit index; *GFI =* goodness of fit index; *RMR =* root-mean square residual; *RMSEA =* root mean square of approximation

Due to this, we then conducted EFA to establish the factor structure of the 28-item Brief COPE. We repeatedly conducted EFA and deleted 7 items including items 1, 6, 9, 12, 17, 26 and 28 since they were loaded on two factors or had a factor loading of less than 0.3 [34]. Two items were also deleted since they distributed among two factors, which caused difficulties in the interpretation. The final EFA results yielded 5-factor structures accounting for 68.26% of the total variances (Table 2). CFA was used to confirm the factor structure obtained from EFA. The Indonesian Brief COPE finally resulted in 5-factors composed of 21-items. The Indonesian Brief COPE 21-item inventory had good model fit with CFI = 0.952, NFI = 0.950, GFI = 0.953, RMR = 0.027, and RMSEA = 0.075. The goodness of fit indices all met the criteria of the cut off values for supporting evident for a fit model. Hence, the Indonesian version of Brief COPE inventory with 5-factor models is acceptable. The following discussion of the Indonesian Brief COPE will refer to this version.

**Table 2:** 5-factor structure of the Brief COPE based on EFA.

No item	Type of coping strategies	Items					
			F1	F2	F3	F4	F5
3	Denial	I’ve been saying to myself "this isn’t real".	**0.758**	-	-	-	-
4	Substance use	I’ve been using alcohol or other drugs to make myself feel better.	**0.917**	-	-	-	-
8	Denial	I’ve been refusing to believe that it has happened.	**0.912**	-	-	-	-
11	Substance use	I’ve been using alcohol or other drugs to help me get through it.	**0.922**	-	-	-	-
13	Self-blame	I’ve been criticizing myself.	**0.834**	-	-	-	-
16	Behavioural disengagement	I’ve been giving up on the attempt to cope.	**0.874**	-	-	-	-
20	Acceptance	I’ve been accepting the reality of the fact that it has happened.	-	**0.642**	-		
22	Religion	I’ve been trying to find comfort in my religion or spiritual beliefs.	-	**0.911**	-	-	**-**
24	Acceptance	I’ve been learning to live with it.	-	**0.704**	-	-	**-**
27	Religion	I’ve been praying or meditating.	-	**0.933**	-	-	**-**
10	Instrumental support	I’ve been getting help and advice from other people.	-		**0.814**		-
15	Emotional support	I’ve been getting comfort and understanding from someone.	-		**0.814**		-
23	Instrumental support	I’ve been trying to get advice or help from other people about what to do.	-		**0.783**		-
5	Emotional support	I’ve been getting emotional support from others.	-		**0.608**		-
7	Active coping	I’ve been taking action to try to make the situation better.	-	**-**	**-**	**0.878**	-
14	Planning	I’ve been trying to come up with a strategy about what to do.	-	**-**	**-**	**0.799**	-
25	Planning	I’ve been thinking hard about what steps to take.	-	**-**	**-**	**0.627**	-
2	Active coping	I’ve been concentrating my efforts on doing something about the situation I’m in.	-	**-**	**-**	**0.610**	-
21	Venting	I’ve been expressing my negative feelings.	-	**-**	**-**	-	**0.842**
18	Humour	I have been making jokes about it					**0.793**
19	Self-distraction	I have been doing something to think about it less such as going to movies, watching TV, reading, day dreaming, sleeping or shopping					**0.553**

### Descriptive statistics

The descriptive statistics (median, actual range) of the Indonesian Brief COPE are shown in Table 3. Regarding the socio-demographic and clinical characteristics, the Indonesian Brief COPE differed on timeframe of diagnosis, with patients who were diagnosed more than 6 months previously having higher coping and patients with higher levels of depression having lower coping (Table 4).

**Table 3:** Descriptive statistics, Cronbach alpha for the major study variables and differences between the Indonesian Brief COPE.

Variable	Median	Actual range	Possible range	Alpha
1. Avoidance coping	10.19	6–24	0–24	0.94
2. Religion and acceptance coping	13.30	4–16	0–16	0.85
3. Social support coping	13.04	4–16	0–16	0.79
4. Problem Solving Coping	11.62	4–16	0–16	0.77
5. Distraction Coping	5.83	3–12	0–12	0.59
6. The Indonesian Brief COPE	54.72	28–90	0–92	0.82
7. DASS-D	5.17	0–40	0–42	0.92
8. DASS-A	5.87	0–36	0–42	0.88
9. DASS-S	7.39	0–38	0–42	0.92
10.Total DASS	18.41	0–112	0–126	0.94
11. FACT-G	63.73	31–105	0–108	0.82

*Note*. *DASS-D =* Depression Anxiety Stress Scale-Depression; *DASS-A =* Depression Anxiety Stress Scale-Anxiety

*DASS-S =* Depression Anxiety Stress Scale-Stress

**Table 4:** Differences in the study variables between depression level group (normal, mild, moderate, severe, very severe).

Variable	normal	mild	moderate	severe	Very severe	F value^a^	P value
	Mean	Mean	Mean	Mean	Mean		
1. Avoidance coping	10.53	10.97	15.22	11.40	12.85	3.313	0.011
2. Religion and acceptance coping	13.58	11.83	12.24	12.50	11.57	5.620	<0.001
3. Social support coping	13.28	11.89	12.00	12.50	11.28	4.394	0.001
4. Problem solving coping	11.86	10.33	10.60	11.10	10.28	5.174	<0.001
5. Distraction coping	5.87	5.25	5.84	6.20	5.85	1.111	0.351
6. Indonesian Brief COPE	55.17	50.27	55.80	53.70	51.85	2.653	0.033

*Note*.

^a^ANOVA Test

## Reliability estimate

### Internal consistency

In the current study, the Cronbach’s alpha coefficient for the Indonesian Brief COPE was 0.82 and its scales ranged from 0.94 to 0.60, which was higher than the original Brief COPE scale [11]. In addition, the coefficients of Cronbach’s **α**-if-item-deleted were all close to the whole scale and subscales indicating good consistency among all items.

### Item total and inter-item correlation

The Indonesian Brief COPE has a variety of corrected item total correlations. The correlations ranged from 0.268 to 0.506. Specifically, item 19 was found to have lower corrected item-total correlation (0.268). However, the Cronbach **α**-if-item-deleted for this item only increases the reliability by 0.002 points which means that deleting this item would not improve the score substantially. The inter item correlations of this inventory were smaller than 0.08 indicating lack of multicollinearity.

### Test and test reliability

Test and retest as represented by intra-class correlation coefficient and Pearson’s correlation were 0.99 and 0.98 respectively. These results purpose that the Indonesian Brief COPE has good stability over a two week period.

## Validity estimate

### Construct validity

Convergent validity was implied by statistically significant and positive correlation between the Indonesian Brief COPE and QoL (r = 0.618, p <0.01). Moreover, the divergent validity was evidenced by statistically significant negative correlation between the Indonesian Brief COPE with depression (r = -0.120, p <0.01), anxiety (r = -0.111, p <0.01) and stress (r = -0.076, p <0.01). All correlations among the major study variables are given in Table 5.

**Table 5:** 

	1	2	3	4	5	6	7	8	9	10	11
1. Avoidance coping	1										
2. Religion and acceptance coping	-0.135**	1									
3. Social support coping	-0.139**	0.601**	1								
4. Problem solving coping	-0.107**	0.561**	0.541**	1							
5. Distraction coping	0.097**	0.369**	0.283**	0.264**	1						
6. The Indonesian Brief COPE	0.609**	0.578**	0.564**	0.562**	0.518**	1					
7. Depression numeric	0.112**	-0.206**	-0.256**	-0.198**	-0.065**	-0.120**	1				
8. Anxiety numeric	0.130**	-0.205**	-0.290**	-0.181**	-0.060**	-0.111**	0.822**	1			
9. Stress numeric	0.152**	-0.171**	-0.243**	-0.179**	-0.075**	-0.076**	0.840**	0.827**	1		
10. Total DASS	0.142**	-0.198**	-0.283**	-0.201**	-0.078**	-0.108**	0.926**	0.916**	0.942**	1	
11. Quality of Life	0.665**	0.147**	0.083**	0.125**	0.292**	0.618**	-0.087**	-0.064**	-0.063**	-0.063**	1

Note.

** Correlation is significant at the 0.01 level (2-tailed).

Contrasted group comparison was also explored. Results showed that the Indonesian Brief COPE and its subscales had significant difference between the different levels of depression groups (normal, mild, moderate, severe and very severe) except for distraction scale indicating this inventory and its scales may significantly discriminate between people who have different levels of depression as shown in Table 4.

## Discussion

This was the first study to evaluate the psychometric properties of the Indonesian Brief COPE. We found a new structure validated in a large sample of ACP with a smaller number of factors. Our results largely supported 5-factor solutions matching most of the original subscales. Furthermore, this Indonesian Brief COPE showed associations and expected relationships with emotional symptoms (stress, anxiety and depression) and QoL, as well as differentiation between the coping strategies used among the different level depression groups in this study.

In this study, we found a 5-factor structure with a total of 21 items resulting in 13 of the 14 coping strategies present in the original Brief COPE. We removed one item from the behavioural disengagement subscales (item 6), one item from the venting subscales (item 21), one item from the self-blame subscales (item 26), and two items from the positive reframing subscales (items 12 and 17) since these items cross-loaded on multiple factors and had item loading of less than 0.30. We also decide to delete item 28 (humour) and item 1 (self-distraction) since humour and self-distraction were distributed among two factors, which caused difficulties in the interpretation. The positive reframing subscale, which refers to ACP trying to see their situation from a different light and looking for a positive experience to evolve, did not appeared in our analysis. These dissimilarities may result from differences in culture, especially religious beliefs. The predominant religion in Indonesia is Islam and many citizens are devout practitioners. Belief in Islam helps patients accept their diagnosis and change their perspective of the world by finding meaning in being a patient with advanced cancer rather than reframing their minds.

The Indonesian Brief COPE inventory categorises five coping structures, namely avoidance coping, religion and acceptance, social support coping, problem solving and distraction. This 5-factor solution is in line with the assumption that the two and three dimensional models of coping might be too simplistic [35]. Furthermore, our results showed that the Brief COPE can be used to measure coping styles in agreement with previous research which described that five coping categories demonstrate the core of coping including problem-solving, support seeking, avoidance, distraction and positive cognitive restructuring [36].

The first factor “avoidance coping” consists of denial, substance use, behavioural disengagement and self-blame. This finding is similar to the previous study [35] that considered these four strategies to be part of a second order coping strategy of avoidance. Some literature considers that avoidant is a kind of passive coping strategies [13]. In this study, avoidant coping had negative correlation with stress, anxiety and depression. These results correspond to the previous research that found avoidant coping accounted for 19–54% of the contributing factors’ effects on the distress variables [37]. However, some literature also claims that sometimes the use of avoidance strategies can help ACP to gradually recognise the threat of cancer and its accompanying consequences which might have a positive effect on individuals when the situation is out of their control [38]. This distinction is primarily due to the strategies being either adaptive or maladaptive, which depend on the context of the patient’s condition, time elapsed and individual resiliency.

The second factor found in our study is “religion and acceptance”. Religion and acceptance gathered in one factor and correlate positively with QoL. Religious coping has been identified by previous research to be the most prevalent coping strategy used by ACP in Indonesia [19]. Belief in Islam helps patients accept their diagnosis. Muslims believe that God’s power controls their health status; this belief helps them to accepted their limitation as humans [39]. In western countries, religiosity is often perceived as a *shared* system of organized beliefs and practises that involves a higher power [40]. Muslims’ definition of religiosity, however, is perceived in a broader and more flexible way, where religion serves as a roadmap to one’s ultimate purpose in life and to a continuous relationship with God [41]. Consequently, the flexibility of religious values in Islam may allow patients to more easily incorporate such values into their coping mechanisms which might influence their quality of life. Additionally, for many Indonesians submission to God (pasrah) is an essential virtue that guides social interactions and maintains harmony. This is thought to potentially reflect the resilient nature of Indonesian culture [39, 42], as gratitude for God even during hard times, along with genuine acceptance of one’s condition, helps Indonesian people endure hardship and achieve emotional wellbeing [39]. This kind of coping helps ACP to have higher resilience to cope with their harsh situation.

The third factor, “social support coping”, includes two conceptually distinct coping mechanisms which use instrumental support and emotional support. This result is similar to previous studies on Indian HIV patients and Greek adults [43, 44]. Theoretically, instrumental support coping is used to get help and advice from other people about what to do, while emotional support involves obtaining comfort, emotional support and understanding [11]. These two types of support principally occur together establishing social support factors that emerge in factor analysis [43]. Strong family bonds are part of the Indonesian culture, unlike in Western cultures, where independence is highly valued and people usually only seek help when absolutely necessary [10].

The fourth factor, “problem solving”, consists of active coping and planning. This result is similar to the finding of [13] which proved that the use of problem-solving strategies would increase quality of life and decrease stress, anxiety and depression. Consistent with our study, the use of problem-solving coping strategies appears to augment the advanced cancer patients’ QoL. In addition, interventions that focus on constructing problem-solving strategies have been identified as recommended treatment in order to increase the quality of life among ACP [45].

“Distraction” is our fifth factor. It consists of venting, self-distraction and humour. Venting refers to expressing unpleasant feelings. Self-distraction focuses on doing something to think less about one’s difficult situation, while humour involves making jokes or making fun of a situation [46]. Humour is often used as it enables the discussion of taboo experiences and makes fun of cancer experiences and its consequence [47]. Basically, these three items aim to temporarily distract ACP’s stress or release stress through transferring or expressing their emotions. Given this, we named our fifth factor distraction coping. In our study, we found this factor to positively correlate with QoL and to be negatively related to stress, depression and anxiety. This might result from distraction strategies and the expression of emotions helping to temporarily deal with physical and emotional symptoms related to their disease progress, which may protect individuals from developing distress by shifting their focus, while, however, leaving the symptoms untreated [48, 49]. Sometimes the same strategy can be adaptive under one condition and maladaptive under another depending partly on the population under study [48]. Additional research is needed on the impact of personality characteristics on the utilization of particular coping strategies.

This study used multiple approaches to examine convergent, divergent and contrast group comparison validities. Results showed that ACP who had lower coping strategies were at higher risk of having depression, stress and anxiety. This finding supports divergent validity as well as higher QoL indicating convergent validity. In addition, contrast group comparison proved that coping strategy scores significantly differentiated ACP who have different levels of depression. In line with this study, the previous research found that anxiety, stress, depression and QoL are strongly associated with coping [6, 14].

Results from CFA suggested that the Indonesian Brief COPE in a sample of ACP has fulfilled the model fit indices. All of the indices were good. According to these base indices, this study has an acceptable fit to the 5 factors models.

### Limitations

This study had several limitations. First, the study was carried out only with ACP at one general referral hospital in one province in Indonesia where patients’ coping may be different than in other provinces. A sample of patients from different provinces should be further recruited to extend generalizability for future studies. Second, at the time of study, most of patients were from outpatient clinics and most of them had a Karnofsky performance scale of at least 60 which means that the participants could manage minimal self-care. Further research should be conducted to explore the coping strategies in this population.

### Implication

The validation of the Indonesian Brief COPE has important implications for clinical practice and research related to coping among ACP in Indonesia. Now a comprehensive assessment which incorporates coping strategies can be utilized to identify coping strategies used by ACP. In addition, assessing coping at the appropriate level of detail can assist health professionals to determine which coping strategies are situationally appropriate. Based on this evidence, new policy guidelines might be tailored to strengthen proper coping and prevent ACP from developing mental health problems. Furthermore, the Indonesian Brief COPE could be used in experimental studies to assess the effectiveness of interventions aimed at supporting appropriate coping strategies.

## Conclusion

This study is the first study to determine factor structure and evaluate the model fit of Brief Cope among an Indonesian population. The results of this study demonstrate that the Indonesian Brief COPE has satisfactory validity and reliability for assessing coping strategies among ACP in Indonesia. The Indonesian Brief COPE is linguistically and culturally equivalent to the original English version. As a valid and reliable questionnaire, it is ready for use in the population to assess coping among native Indonesian–speaking advanced cancer patients.
